# Childhood abuse and adult cyberbullying victimization in Canada: The role of community belonging

**DOI:** 10.1371/journal.pone.0337552

**Published:** 2025-12-08

**Authors:** Emily Earle, David Speed

**Affiliations:** Department of Psychology, University of New Brunswick, Saint John, New Brunswick, Canada; Australian Catholic University, AUSTRALIA

## Abstract

Childhood abuse is linked to a higher risk of experiencing cyberbullying victimization in adolescence and young adulthood, while a sense of belonging is associated with a reduced risk and buffers against the mental health effects of childhood trauma. However, there is a lack of Canadian research, particularly studies involving adult samples, examining the connection between childhood abuse and cyberbullying victimization in adulthood. The current study examined whether community belonging is a protective factor in the association between childhood abuse and cyberbullying victimization among adults. Data came from Cycle 34 of the Canadian General Social Survey (*n* ≈ 14,000). Respondents were asked about the frequency in which they experienced seven childhood abuse variables and about the strength of their current community belonging. A binary logistic regression with bootstrap and person-level weights was conducted, while controlling for demographic variables. Results showed that: 1) for each childhood abuse variable, never experiencing that behaviour was associated with trivial-to-small effects for lower cyberbullying victimization in adulthood compared to those who experienced high levels of that behaviour; 2) as community belonging increased, the odds of adult cyberbullying victimization decreased; and 3) there were no significant moderating effects of community belonging; however, a pattern emerged suggesting that differences in cyberbullying victimization between those who did not experience a given abusive behaviour in childhood and those who did experience high levels of that behaviour were larger when community belonging was weaker and smaller when belonging was stronger. The current study highlights the importance of fostering community connections, for example, through programs, support groups, and volunteering, as a potential way of reducing the risk of cyberbullying among adults who have experienced childhood abuse.

## Introduction

Child maltreatment, including abuse, neglect, and exploitation of an individual under the age of 18 by a person in a position of authority or trust, is positively associated with cyberbullying victimization (CBV), particularly during adolescence [1–8]; however, more research is needed to examine this association in adulthood. Cyberbullying, defined as bullying that occurs via electronic technology, can take many forms, such as harassment, trickery, exclusion, and impersonation, and it can occur on different media platforms, including email, text messages, chat rooms, and online games [9,10]. Technology use is increasing across all age groups [11], with adolescents and young adults being more proficient internet users [12]. As younger, more proficient cohorts age, CBV rates in middle-to-late adulthood may rise [13], suggesting a greater need to study factors that mitigate the risk of CBV in adulthood. Research on protective factors against CBV among individuals with a history of childhood abuse remains limited [6], although a sense of belonging has been shown to protect against the long-term psychological effects of childhood abuse [14–16]. Drawing on the social ecological framework [17], the current study examined whether community belonging mitigates CBV risk in adulthood in the context of adult recollections of past childhood abuse, which can inform intervention strategies.

### Childhood victimization

Childhood physical abuse (CPA), childhood sexual abuse (CSA), and childhood emotional abuse (CEA) are frequent early-life stressors. CPA involves unreasonable force inflicted on a child's body, either by an adult or an older youth [18]. CSA includes any sexual act by an adult or youth towards a child (under the age of 16) for sexual gratification [18]. CEA is the continual exposure to behaviours that negatively affect a child’s psychological, emotional, or spiritual development by an adult [18]. In 2018, 24.0% of Canadians reported having experienced CPA, 7.8% experienced CSA, and approximately 30.0% experienced CEA before age 15 [19,20].

### Cyberbullying victimization

Higher internet use across all age groups in Canada is correlated with higher CBV risk [11]. However, past research on CBV has primarily focused on children and adolescents [21], with less attention on adults, especially over the age of 30. In 2019, 25% of Canadians aged 12–17 reported CBV in the previous year, and in 2018, 25% of young adults in Canada aged 18–29 reported it [12]. General trends show that CBV is highest in adolescence and young adulthood, gradually decreasing by the late 20s [12]. In 2014, 6.1% of Canadians aged 26–40, 3.7% aged 41–60, and 1.7% aged 61 and older experienced some form of cyberbullying in the past five years [22]. Therefore, CBV remains a relevant issue in middle-to-late adulthood.

Several factors predict increased CBV risk. Young women (32%) experience CBV more than young men (17%), with non-binary individuals facing even higher rates [11,12]. Additional risk factors include same-gender attraction, disability, low social skills, limited social support, and poor emotion regulation, along with mental health conditions, which can be both a cause and consequence of CBV [12,22,23]. Furthermore, adverse childhood experiences also have been linked to CBV [7]. Given that CBV is associated with poor mental health outcomes [21,22], it is important to better understand how these early risk factors contribute to this form of victimization.

### Childhood victimization and CBV

Childhood victimization, including CPA, CSA, and CEA, has been associated with a heightened vulnerability to experiencing subsequent victimization later in life [24]. Consistent with this literature, recent research examining child, adolescent, and young adult samples has found a positive correlation between childhood abuse and experiencing cyberbullying [1,2,7]. Moreover, previous literature indicates that the effects of early abuse persist across developmental stages [25]; therefore, such experiences may also impact CBV risk in adulthood.

The mechanism connecting childhood abuse with CBV likely involves developmental and mental health-related pathways. Bronfenbrenner’s ecological systems theory provides a framework for understanding how childhood experiences shape development and later outcomes [17] and has been applied to explain both childhood abuse [26] and bullying [27,28]. The theory posits that the individual is at the centre as an active participant and that development occurs within four interconnected systems: the microsystem (immediate environments such as home, school, and peers), mesosystem (interactions among the microsystems), exosystem (environments that indirectly affect the child), and macrosystem (the broader cultural and societal context). These systems are situated within the chronosystem, which reflects changes over time. Together, the systems represent progressively more distant influences on the individual [17].

Some research has used the ecological systems model to explain the connection between childhood abuse and different forms of revictimization later in life [29,30]. A similar framework has been used to explain the connection between childhood abuse and CBV [6]. At the individual level, childhood abuse has been shown to impair social skills, emotional regulation, and self-esteem [6,31,32]. These impairments may contribute to maladaptive coping strategies and difficulties in interpersonal relationship [33,34], thus increasing vulnerability to CBV in adulthood. At the microsystem level, families and peers exert direct influence on children. For instance, children raised in homes with abuse, aggression, or interparental violence may come to perceive such behaviour as normal, which can impair the ability to form healthy peer relationships and increase their acceptance of bullying victimization [2,28]. Furthermore, children and adolescents who experience abuse may have fewer restrictions on technology, and these habits may persist into adulthood, increasing the risk of CBV [7]. Notably, research on the mesosystem in the context of revictimization is limited. One example could be conflict between a parent and the child's teachers, which may increase the child's stress, reduce the level of support they receive at school, and exacerbate lasting emotional difficulties. At the exosystem level, social media and internet use can influence CBV risk. Adverse childhood experiences and harsh parenting have been linked to increased smartphone and internet use in adolescence and young adulthood, increasing risk of CBV [35,36], which may persist throughout adulthood. At the macrosystem level, broader societal views influence how individuals later perceive and respond to victimization. The social acceptance of physical punishment as a normative form of discipline in Canada [37], poorer recognition of emotionally abusive behaviours [38], and stigma surrounding sexual abuse that discourages disclosure and help-seeking [39] may contribute to the normalization of aggression. Within the ecological systems framework, protective factors such as community cohesion have been studied in the context of revictimization [40], suggesting that a sense of community belonging may also be protective.

### Sense of belonging

An individual’s perceived sense of belonging in a community may influence CBV vulnerability. Community belonging encompasses an individual’s comfort, social connections, and participation within their community [41]. In 2022, 47% of Canadians reported a strong sense of community belonging, which varied across age categories. Individuals aged 15–34 reported the weakest sense of belonging, while those aged 65 and older reported the strongest [42]. Research also shows that people living in rural areas report higher levels of community belonging than people living in urban areas [41]. Furthermore, a sense of community belonging is correlated with better mental health outcomes across all age groups [43]. Considering its impact on support and mental health, community belonging may buffer the impact of childhood victimization on CBV in adulthood.

### Childhood victimization, CBV, and a sense of belonging

Although childhood abuse is associated with later CBV, not all individuals who experience abuse are affected in the same way. Resilience factors exist at each level of the ecological framework [44], with research showing that general resiliency outcomes are higher among maltreated children who have stronger community support and cohesion [44,45]. Moreover, belonging to peer, family, and community groups buffers the effects of childhood trauma on mental health, risky, substance use, and well-being [16,46], and may similarly reduce the risk of CBV. Attachment Theory [47] suggests that when a child is abused by a trusted adult, their sense of secure attachment is impaired [48], which negatively affects that individual’s ability to engage in healthy social relationships [16,49]. Conversely, a sense of belonging may foster social skills, self-confidence, and self-esteem, while also encouraging community engagement and reducing time on social media, potentially lowering the risk of CBV [27]. However, its role in the link between childhood abuse and CBV remains unclear.

### Current study

There are three main gaps in the literature exploring the relationship between childhood abuse and CBV. First, most research on this topic has been done outside of Canada and has focused on adolescents rather than adults. Second, prior studies have examined aggregated childhood abuse rather than individual subtypes [6]. Third, there are limited studies examining potential factors that may mitigate the risk of CBV among individuals with a history of childhood abuse. To bridge these gaps, the current study used the 2019 General Social Survey (Cycle 34) to: 1) examine whether those who experienced childhood abuse were at an increased risk of CBV as adults, and 2) determine whether a sense of community belonging buffered the relationship. The research questions and hypotheses were as followed:

**RQ1:** Does experiencing CPA, CSA, or CEA predict increased odds of CBV in adulthood?
  **H1:** Individuals who recalled having experienced CPA, CSA, or CEA at lower levels will have lower odds of CBV in adulthood than individuals who experienced higher levels of abuse in childhood.
**RQ2:** Do the odds of CBV in adulthood decrease with increased community belonging?
  **H2:** Community belonging will negatively predict adult CBV.
**RQ3:** Does a sense of community belonging buffer the relationship between childhood abuse and adult CBV?
  **H3**: A sense of community belonging will moderate the relationship between recollections of childhood abuse and the odds of CBV in adulthood, such that those who report experiences of childhood abuse but have stronger current community belonging will have lower odds of experiencing CBV in adulthood compared to those with lower belonging. In other words, the negative slope of community belonging and abuse will become flatter for non-abused respondents.

## Method

### Data

We accessed the Master file of the 2019 General Social Survey (GSS; Cycle 34), a cross-sectional survey on victimization in Canada [50]. The two main objectives of the GSS are to monitor changes in Canadians’ living conditions and well-being and to provide information on social policy issues. The 2019 GSS collected data from April 2019 to March 2020 using telephone and electronic questionnaires in the provinces. In the territories, computer-assisted interviews were used as well. The target population was persons 15 years of age or older living in Canada, excluding full-time residents of institutions. The 2019 GSS stratified the ten provinces by geographic area and created a survey frame using the telephone numbers available to Statistics Canada and a list of dwellings within the ten provinces (territories were not included due to methodology differences). For each stratum, a random sample without replacement was selected (response rate was 36.4%). To be included in the current study, respondents had to be 19 years or older, have answered all questions of interest, and reside in the provinces (i.e., those living in the territories were excluded). The 2019 GSS is the most recent wave of that survey to examine all predictors and outcomes of interest (see Table 1 for descriptive statistics).

**Table 1:** Weighted Descriptive Statistics for Covariates, Childhood Abuse, Belonging, and Cyberbullying.

Variable	Total Sample (*n* ≈* *14,000)
Income	5.60/7.95
Age	47.30/17.01
Married/Common Law	65.6%
Widowed/Separated/Divorced	11.0%
Single	23.3%
Female	50.8%
Heterosexual	96.4%
Atlantic	6.70%
Quebec	23.0%
Ontario	38.5%
Prairies	18.1%
British Columbia	13.8%
Born Outside of Canada	28.2%
Born in Canada	71.8%
White and non-Aboriginal	72.8%
White and Aboriginal	2.8%
Non-White and non-Aboriginal	24.4%
≤ High school	28.1%
College or trade school	36.8%
Bachelor’s or higher	35.1%
Urban Population Centre	83.4%
Rural and Prince Edward Island	16.6%
CPA-Slapped 6+ times	6.8%
Slapped 1–5 times	14.1%
CPA-Pushed 6+ times	3.5%
Pushed 1–5 times	7.5%
CPA-Kicked 6+ times	1.7%
Kicked 1–5 times	3.4%
CSA-Forced 6+ times	1.0%
Forced 1–5 times	2.5%
CSA-Touched 6+ times	1.5%
Touched 1–5 times	5.2%
CEA-Felt Hurt 6+ times	16.5%
Felt Hurt 1–5 times	25.4%
CEA- Felt Unloved 6+ times	8.4%
Felt Unloved 1–5 times	9.8%
Cyberbullied in Past 5 Years	8.8%
Community Belonging	2.85/0.89

*Note.* CPA = childhood physical abuse; CSA = childhood sexual abuse; CEA = childhood emotional abuse. Percentages in the “never” categories can be calculated. Cells may not equal 100% due to rounding. Categorical variables are presented as mean/standard deviation. Sample size is rounded to the nearest thousand in accordance with Statistics Canada’s guidelines to reduce the risk of disclosure.

The authors obtained access to a secure research data centre, where all analyses were conducted. Results were vetted by Statistics Canada staff to ensure that respondents from the GSS could not be identified. As part of Statistic Canada’s guidelines, random digit rounding was used, where the sample size was rounded to the nearest thousand. This does not affect statistical reporting, only the presented descriptive values and their respective sample size. We did not seek approval from the Research Ethics Board for the current study because the use of data from Statistics Canada falls under an exception in the Tri-Council Policy Statement Section, Article 2.2 [51].

### Measures

#### Covariates.

We included the following as covariates in the current study: age, age^2^, income (increments of $10,000), biological sex (female = reference, male), sexual orientation (heterosexual = reference, non-heterosexual), marital status (married/common-law = reference, widowed/separated/divorced, single), highest educational attainment (high school or less = reference, college, trade school diploma or less than bachelors, bachelor’s or higher), region (Atlantic = reference, Quebec, Ontario, Prairies, British Columbia), ethnoracial identity (White and non-Aboriginal = reference, White and Aboriginal, non-White and non- Aboriginal), place of birth (Outside of Canada = reference, Canada), and population centre type (urban = reference, rural and Prince Edward Island) [52]. These covariates were selected based on previous research, which often used child or adolescent samples and controlled for parental information [2,7]; in the current study, focused on adults, adult measures were used. Covariates were also informed by studies examining predictors of community belonging [41] and CBV [12].

#### Childhood abuse.

The 2019 GSS included three questions about CPA, two about CSA, and two about CEA [52]. To measure CPA respondents were asked, “Before age 15, how many times did an adult do any of the following”: (1) “Slap you on the face, head or ears or hit you with something hard to hurt you”; (2) “Push, grab, shove or throw something at you to hurt you”; and (3) “Kick, bite, punch, choke, burn you, or physically attack you in some way”? To measure CSA, respondents were asked, “Before age 15, how many times did an adult do any of the following: (1) “Force you or attempt to force you into unwanted sexual activity, by threatening you, holding you down or hurting you in some way,” and (2) “Touch you against your will in any sexual way” (Statistics Canada, 2019)? To measure CEA, respondents were asked, “Before age 15, how many times did you parents and other caregivers do any of the following: (1) “Say things that really hurt your feelings,” and (2) “Made you feel like you were not wanted or loved” [52]. In the current study, responses were recoded to be 0 (*never experiencing it*), 1 (*experiencing it one to five times),* and 2 (*experiencing it six or more times*). These cut-points were chosen to maximize the number of individuals in groups. The reference group was the six or more times category, with groups labeled as ‘Never-abuse’, ‘Low-to-moderate abuse’, and ‘High-abuse’. In total, we had seven abuse variables with three levels each.

#### Sense of community belonging.

The 2019 GSS asked, “How would you describe your sense of belonging to your local community?” [52]. Response options included 1 (*very weak*), 2 (*somewhat weak*), 3 (*somewhat strong*), and 4 (*very strong*). The sense of community belonging outcome was treated as a continuous variable ranging from low-to high-belonging.

#### Cyberbullying.

The outcome of interest in the current study was adult CBV. The 2019 GSS included five questions on experience with specific types of CBV and a derived variable measuring whether respondents were cyber-stalked or cyberbullied in the last five years [52]. The derived variable (No = reference, Yes) was used as the outcome in the current study.

### Data analysis

All data analyses were conducted using Stata 15 software [53]. A binary logistic regression model was conducted with bootstrap and person-level weights, while adjusting for covariates. Pseudo-*R*^2^ values could not be estimated due to the use of these weights. All analyses were assessed using an α level of.05. We explored effect sizes in the context of odds ratios (*OR*). An *OR* of 1.44 (or 0.70 for a negative association) was the threshold for a small effect, 2.48 (or 0.40) was the cutoff for a medium effect, and 4.27 (or 0.23) was the threshold for a large effect [54]. The block structure was as follows:

**Block 1:** Demographic covariates were entered.
**Block 2:** Predictors (i.e., CPA, CSA, and CEA variables) were entered.
**Block 3:** Sense of community belonging was entered.
**Block 4:** Interaction terms between belonging and CPA variable one (i.e., belonging * Slap CPA) were explored.
**Block 5:** Interaction terms from Block 4 were removed; interactions between belonging and CPA variable two (i.e., belonging * Push CPA) were explored.
**Block 6:** Interaction terms from Block 5 were removed; interactions between belonging and CPA variable three (i.e., belonging * Kick CPA) were explored.
**Block 7:** Interaction terms from Block 6 were removed; interactions between belonging and CSA variable one (i.e., belonging * Force CSA) were explored.
**Block 8:** Interaction terms from Block 7 were removed; interactions between belonging and CSA variable two (i.e., belonging * Touch CSA) were explored.
**Block 9:** Interaction terms from Block 8 were removed; interactions between belonging and CEA variable one (i.e., belonging * Hurt CEA) were explored.
**Block 10:** Interaction terms from Block 9 were removed; interactions between belonging and CEA variable two (i.e., belonging * Unloved CEA) were explored.

In our models, between one and four bootstrap replications could not be estimated (out of 500), although this did not indicate any substantive issues. These issues occurred because sampling weights provided by Statistics Canada did not include enough respondents to estimate the model.

## Results

In Block 1, adult CBV was regressed on covariates, and the block was significant, *F*(17, 500) = 4.47, **p* *< .001. Specifically, being widowed, separated, or divorced, being single, identifying as non-heterosexual, being White and Aboriginal, obtaining a college or trade school certificate, and holding a bachelor’s degree or higher all predicted significantly higher odds of CBV in adulthood compared to their respective reference groups (see Table 2). In Block 2, the childhood abuse predictors were added, and the block was significant, *ΔF(*14, 500) = 6.25, **p* *< .001, but the ‘Never-abuse’ categories and the ‘Low-to-moderate abuse’ categories did not differ significantly from their base groups, failing to support H1. A sense of community belonging was added in Block 3, and the overall block was significant *ΔF(*1, 500) = 6.25, **p* *= .013. As community belonging increased the odds of CBV decreased, *OR* = 0.86, 95% CI [0.77, 0.97], *t *= −2.50, **p* *= .013, which was consistent with H2 (see Table 2).

**Table 2:** Likelihood of Cyberbullying Victimization in the Past Five Years Based on Covariates Childhood Physical, Sexual, and Emotional Abuse, and Community Belonging (*n*  ≈  14,000).

	Odds Ratio [95% Confidence Intervals]		
	Block 1	Block 2	Block 3
Constant	0.03 [0.01, 0.08]	0.15 [0.04, 0.60]^**^	0.20 [0.05, 0.86]^*^
Income	1.00 [0.99, 1.02]	1.01 [0.99, 1.02]	1.01 [0.99, 1.02]
Age	1.03 [0.99, 1.07]	1.02 [0.97, 1.06]	1.02 [0.98, 1.06]
Age^2^	1.00 [1.00, 1.00]	1.00 [1.00, 1.00]	1.00 [1.00, 1.00]
Married/Common Law (ref.)			
Widowed/Separated/Divorced	1.73 [1.36, 2.21]^***^	1.52 [1.17, 1.98]^**^	1.49 [1.14, 1.95] ^**^
Single	1.52 [1.20, 1.92]^***^	1.42 [1.12, 1.81]^**^	1.39 [1.09, 1.77] ^**^
Female (ref.)			
Male	1.07 [0.87, 1.31]	1.10 [0.90, 1.35]	1.09 [0.89, 1.34]
Heterosexual (ref.)			
Non-Heterosexual	1.65 [1.07, 2.53]^*^	1.45 [0.96, 2.19]^*^	1.41 [0.92, 2.14]
Atlantic (ref.)			
Quebec	1.02 [0.75, 1.38]	1.03 [0.76, 1.41]	1.01 [0.74, 1.38]
Ontario	0.96 [0.74, 1.25]	0.93 [0.71, 1.21]	0.93 [0.71, 1.21]
Prairies	1.11 [0.85, 1.44]	1.04 [0.80, 1.35]	1.03 [0.79, 1.35]
British Columbia	0.86 [0.63, 1.17]	0.82 [0.60, 1.12]	0.82 [0.60, 1.12]
Born Outside of Canada (ref.)			
Born in Canada	1.00 [0.74, 1.34]	0.97 [0.72, 1.30]	0.97 [0.72, 1.30]
White and Non-Aboriginal (ref.)			
White and Aboriginal	2.25 [1.37, 3.68]^***^	1.97 [1.17, 3.31]^**^	2.00 [1.20, 3.34] ^*^
Non-White and Non-Aboriginal	1.06 [0.74, 1.52]	1.07 [0.75, 1.53]	1.08 [0.76, 1.54]
≤ High school (ref.)			
College or trade school	1.35 [1.02, 1.79]^*^	1.31 [0.98, 1.75]^†^	1.31 [0.98, 1.74]^†^
Bachelor’s or higher	1.55 [1.16, 2.06]^**^	1.52 [1.14, 2.03]^**^	1.51 [1.13, 2.02] ^**^
Urban Centre (ref.)			
Rural and PEI	0.90 [0.70, 1.17]	0.94 [0.72, 1.22]	0.98 [0.75, 1.27]
CPA-Slapped 6+ times (ref.)			
Never Slapped		0.76 [0.48, 1.21]	0.77 [0.49, 1.23]
Slapped 1–5 times		1.23 [0.79, 1.93]	1.23 [0.79, 1.93]
CPA-Pushed 6+ times (ref.)			
Never Pushed		0.91 [0.50, 1.66]	0.90 [0.49, 1.64]
Pushed 1–5 times		1.13 [0.62, 2.07]	1.13 [0.62, 2.06]
CPA-Kicked 6+ times (ref.)			
Never Kicked		0.68 [0.35, 1.29]	0.68 [0.35, 1.30]
Kicked 1–5 times		0.79 [0.39, 1.59]	0.79 [0.39, 1.60]
CSA-Forced 6+ times (ref.)			
Never Forced		0.74 [0.26, 2.15]	0.75 [0.26, 2.17]
Forced 1–5 times		1.11 [0.35, 3.50]	1.13 [0.36, 3.55]
CSA-Touched Six+ (ref.)			
Never Touched		0.99 [0.39, 2.55]	1.00 [0.39, 2.57]
Touched 1–5 times		1.22 [0.46, 3.26]	1.22 [0.46, 3.26]
CEA-Felt Hurt 6+ times (ref.)			
Never Felt Hurt		0.92 [0.64, 1.32]	0.94 [0.65, 1.35]
Felt Hurt 1–5 times		1.01 [0.72, 1.42]	1.03 [0.73, 1.44]
CEA-Felt Unloved 6+ times (ref.)			
Never Felt Unloved		0.70 [0.46, 1.05]^†^	0.70 [0.46, 1.07]^†^
Felt Unloved 1–5 times		0.95 [0.64, 1.42]	0.95 [0.63, 1.42]
Community Belonging			0.86 [0.77, 0.97]^*^
*∆* *F*	4.47^***^	6.25^***^	6.25^*^

*Note.* Ref. = reference group; CPA = childhood physical abuse; CSA = childhood sexual abuse; CEA = childhood emotional abuse.

^†^ *p* ≤.10, * *p* ≤.05, ** *p* ≤.01, *** *p* ≤.001.

**Fig 1:**
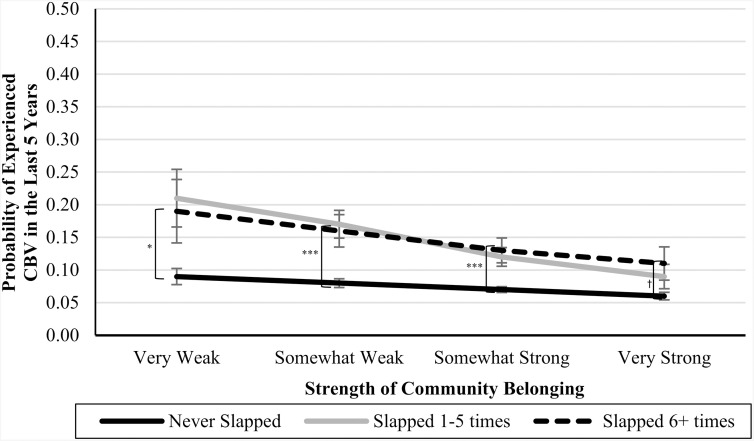
The Interaction Between Being Slapped in Childhood and Current Community Belonging in the Prediction of Cyberbullying Risk in Adulthood. Error bars represent standard error. CBV = Cyberbullying victimization. The left brackets represent the difference in the likelihood of CBV between respondents in the ‘Never-abuse’ group and those in the ‘High-abuse’ group at each level of community belonging. Only significant differences are shown. ^†^ *p* ≤ .10, ^*^ **p* *≤ .05, ^***^ **p* *≤ .001.

**Fig 2:**
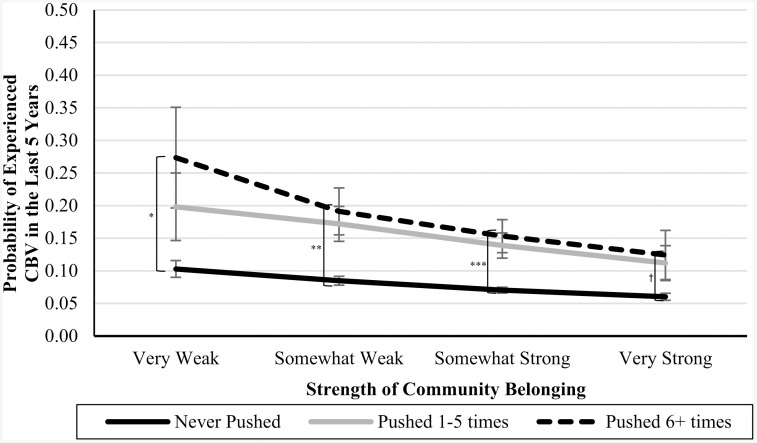
The Interaction Between Being Pushed in Childhood and Current Community Belonging in the Prediction of Cyberbullying Risk in Adulthood. Error bars represent standard error. CBV = Cyberbullying victimization. The left brackets represent the difference in the likelihood of CBV between respondents in the ‘Never-abuse’ group and those in the ‘High-abuse’ group at each level of community belonging. Only significant differences are shown. ^†^ *p* ≤ .10, ^*^ **p* *≤ .05, ^**^ **p* *≤ .01, ^***^ **p* *≤ .001.

**Fig 3:**
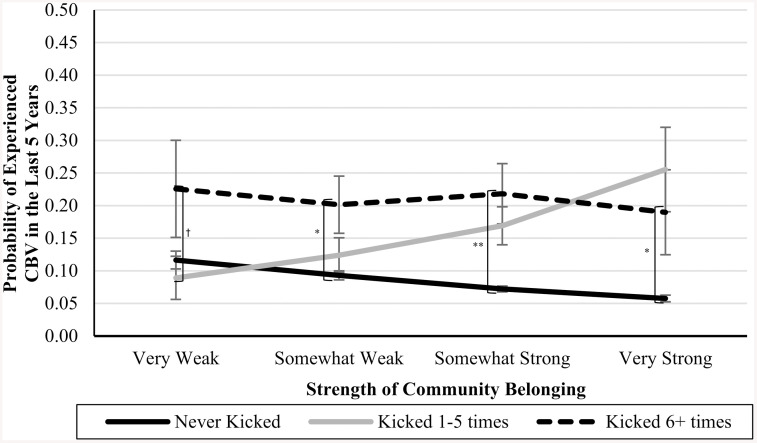
The Interaction Between Being Kicked in Childhood and Current Community Belonging in the Prediction of Cyberbullying Risk in Adulthood. Error bars represent standard error. CBV = Cyberbullying victimization. The left brackets represent the difference in the likelihood of CBV between respondents in the ‘Never-abuse’ group and those in the ‘High-abuse’ group at each level of community belonging. The right bracket represents the difference in the likelihood of CBV between respondents in the ‘Low-to-moderate’ groups and those in the ‘High-abuse’ group at each level of community belonging. Only significant differences are shown. ^†^ *p* ≤ .10, ^*^ **p* *≤ .05, ^**^ **p* *≤ .01.

**Fig 4:**
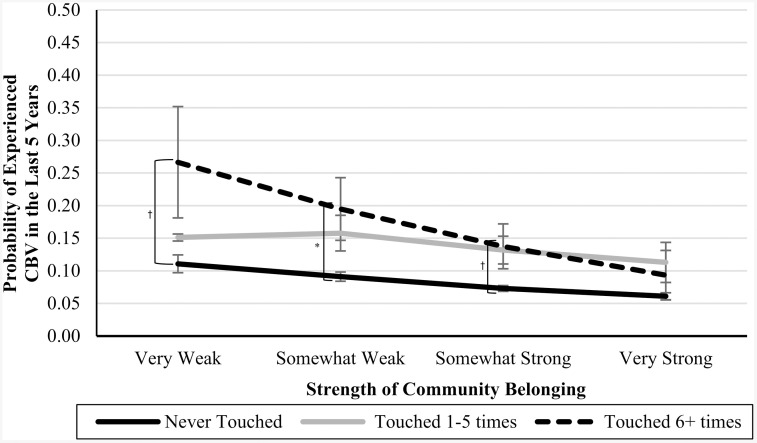
The Interaction Between Being Touched in Childhood and Current Community Belonging in the Prediction of Cyberbullying Risk in Adulthood. Error bars represent standard error. CBV = Cyberbullying victimization. The left brackets represent the difference in the likelihood of CBV between respondents in the ‘Never-abuse’ group and those in the ‘High-abuse’ group at each level of community belonging. Only significant differences are shown. ^†^ *p* ≤ .10, ^*^ **p* *≤ .05.

**Fig 5:**
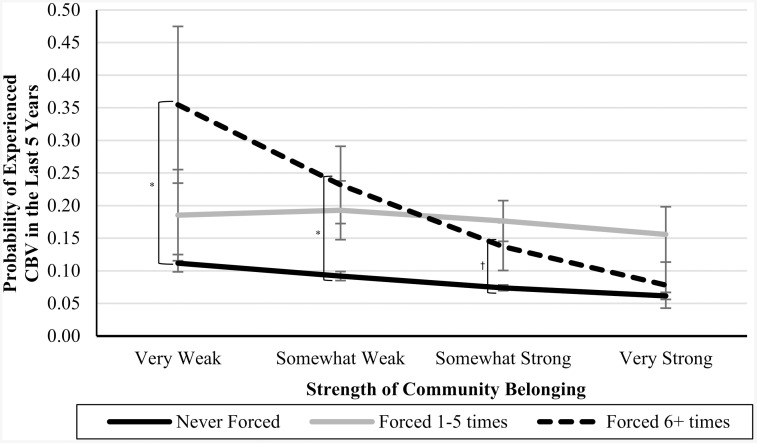
The Interaction Between Being Forced Into Sexual Activity in Childhood and Current Community Belonging in the Prediction of Cyberbullying Risk in Adulthood. Error bars represent standard error. CBV = Cyberbullying victimization. The left brackets represent the difference in the likelihood of CBV between respondents in the ‘Never-abuse’ group and those in the ‘High-abuse’ group at each level of community belonging. Only significant differences are shown. ^†^ *p* ≤ .10, ^*^ **p* *≤ .05.

**Fig 6:**
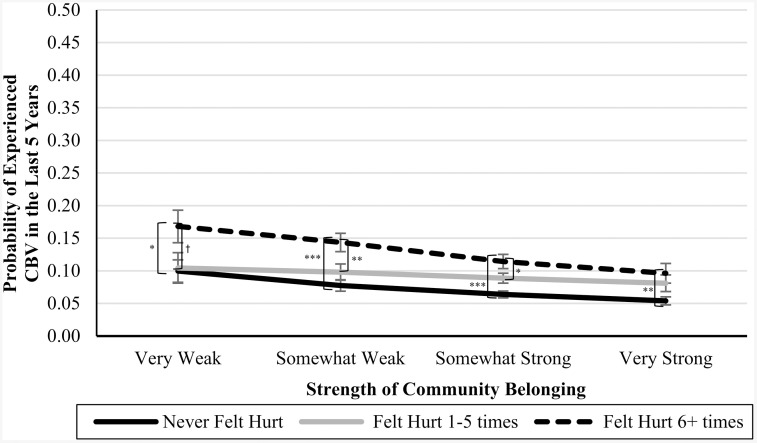
The Interaction Between Feeling Emotionally Hurt in Childhood and Current Community Belonging in the Prediction of Cyberbullying Risk in Adulthood. Error bars represent standard error. CBV = Cyberbullying victimization. The left brackets represent the difference in the likelihood of CBV between respondents in the ‘Never-abuse’ group and those in the ‘High-abuse’ group at each level of community belonging. The right brackets represent the difference in the likelihood of CBV between respondents in the ‘Low-to-moderate’ groups and those in the ‘High-abuse’ group at each level of community belonging. Only significant differences are shown. ^†^ *p* ≤ .10, ^*^ **p* *≤ .05, ^**^ **p* *≤ .01, ^***^ **p* *≤ .001.

**Fig 7:**
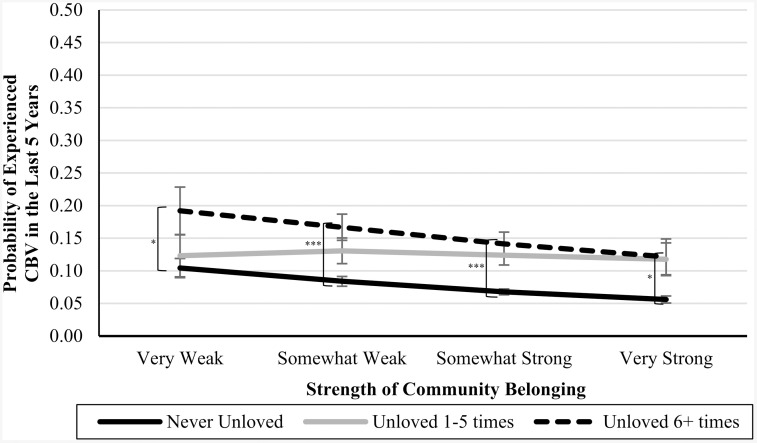
The Interaction Between Feeling Unloved in Childhood and Current Community Belonging in the Prediction of Cyberbullying Risk in Adulthood. Error bars represent standard error. CBV = Cyberbullying victimization. The left brackets represent the difference in the likelihood of CBV between respondents in the ‘Never-abuse’ group and those in the ‘High-abuse’ group at each level of community belonging. Only significant differences are shown. ^*^ **p* *≤ .05, ^***^ **p* *≤ .001.

In Blocks 4–6, we introduced interaction terms between the CPA variables and community belonging (see Table 3). In Blocks 7 and 8, we introduced interaction terms between the CSA variables and community belonging (see Table 4). In Blocks 9 and 10, we introduced interaction terms between the CEA variables and community belonging (also in Table 4). In each of these cases, the interaction terms were not significant predictors of CBV, and the nonsignificant *ΔF*-statistics at the bottom of each column indicate that the models did not significantly improve. However, the marginal means analyses (presented in Table 5), are suggestive of an underpowered interaction effect. See Figs 1–7 for visualizations of the interaction terms.

**Table 3:** Likelihood of Cyberbullying Victimization Based on Covariates, Childhood Physical, Sexual, and Emotional Abuse and Community Belonging, with Interactions Between Childhood Physical Abuse Variables and Community Belonging (*n* ≈ 14,000).

		Odds Ratio [95% Confidence Intervals]	
	Block 4	Block 5	Block 6
Constant	1.01 [0.99, 1.02]^†^	0.28 [0.05, 1.59]	0.14 [0.02, 0.82]^*^
CPA-Slapped 6+ times (ref.)			
Never Slapped	0.63 [0.23, 1.77]	0.77 [0.48, 1.23]	0.75 [0.48, 1.18]
Slapped 1–5 times	1.70 [0.52, 5.55]	1.23 [0.79, 1.94]	1.20 [0.77, 1.86]
CPA-Pushed 6+ times (ref.)			
Never Pushed	0.90 [0.49, 1.65]	0.61 [0.17, 2.25]	0.92 [0.51, 1.67]
Pushed 1–5 times	1.13 [0.61, 2.07]	0.85 [0.18, 4.08]	1.16 [0.64, 2.09]
CPA-Kicked 6+ times (ref.)			
Never Kicked	0.68 [0.35, 1.30]	0.68 [0.35, 1.30]	1.14 [0.30, 4.27]
Kicked 1–5 times	0.78 [0.38, 1.60]	0.79 [0.38, 1.61]	0.23 [0.04, 1.35]
CSA-Forced 6+ times (ref.)			
Never Forced	0.76 [0.26, 2.19]	0.76 [0.26, 2.20]	0.75 [0.26, 2.16]
Forced 1–5 times	1.14 [0.36, 3.61]	1.14 [0.36, 3.61]	1.11 [0.35, 3.48]
CSA-Touched 6+ times (ref.)			
Never Touched	0.99 [0.38, 2.56]	1.00 [0.39, 2.57]	0.99 [0.39, 2.53]
Touched 1–5 times	1.22 [0.46, 3.26]	1.22 [0.45, 3.26]	1.22 [0.46, 3.23]
CEA-Felt Hurt 6+ times (ref.)			
Never Felt Hurt	0.93 [0.65, 1.34]	0.94 [0.65, 1.35]	0.94 [0.65, 1.35]
Felt Hurt 1–5 times	1.02 [0.73, 1.44]	1.03 [0.73, 1.44]	1.02 [0.73, 1.43]
CEA-Felt Unloved 6+ times (ref.)			
Never Felt Unloved	0.71 [0.47, 1.08]	0.71 [0.46, 1.08]	0.70 [0.46, 1.07]^†^
Felt Unloved 1–5 times	0.95 [0.64, 1.43]	0.95 [0.63, 1.43]	0.95 [0.63, 1.42]
Community Belonging	0.85 [0.62, 1.16]	0.76 [0.50, 1.14]	1.01 [0.64, 1.59]
Belonging*Slapped 6+ times (ref.)			
Belonging*Never Slapped	1.08 [0.77, 1.50]		
Belonging*Slapped 1–5 times	0.88 [0.58, 1.35]		
Belonging*Pushed 6+ times (ref.)			
Belonging*Never Pushed		1.16 [0.76, 1.77]	
Belonging*Pushed 1–5 times		1.12 [0.66, 1.91]	
Belonging*Kicked 6+ times (ref.)			
Belonging*Never Kicked			0.82 [0.52, 1.30]
Belonging*Kicked 1–5 times			1.59 [0.85, 2.97]
*∆* *F*	0.82	0.27	4.90^**^

*Note.* Ref. = reference group; CPA = childhood physical abuse; CSA = childhood sexual abuse; CEA = childhood emotional abuse. All models controlled for age, age^2^, income, sex, sexual orientation, marital status, education, region, ethnoracial identity, place of birth, and population centre. Covariates were omitted from the table to improve clarity.

^†^ *p* ≤.10, * *p* ≤.05, ** *p* ≤.01, *** *p* ≤.001.

**Table 4:** Likelihood of Cyberbullying Victimization Based on Covariates, Childhood Physical, Sexual, and Emotional Abuse and Community Belonging, with Interactions Between Childhood Sexual and Emotional Abuse Variables and Community Belonging (*n* ≈ 14,000).

	Odds Ratio [95% Confidence Intervals]			
	Block 7	Block 8	Block 9	Block 10
Constant	0.50 [0.07, 3.59]	0.33 [0.05, 2.02]	0.22 [0.05, 0.98]^*^	0.21 [0.04, 0.97]^*^
CPA-Slapped 6+ times (ref.)				
Never Slapped	0.77 [0.49, 1.23]	0.77 [0.49, 1.23]	0.77 [0.49, 1.24]	0.77 [0.48, 1.22]
Slapped 1–5 times	1.23 [0.79, 1.93]	1.23 [0.79, 1.93]	1.24 [0.79, 1.96]	1.22 [0.78, 1.91]
CPA-Pushed 6+ times (ref.)				
Never Pushed	0.89 [0.49, 1.62]	0.89 [0.49, 1.63]	0.89 [0.49, 1.63]	0.91 [0.50, 1.66]
Pushed 1–5 times	1.11 [0.61, 2.04]	1.12 [0.61, 2.05]	1.12 [0.61, 2.05]	1.15 [0.63, 2.10]
CPA-Kicked 6+ times (ref.)				
Never Kicked	0.69 [0.35, 1.33]	0.68 [0.35, 1.32]	0.68 [0.35, 1.30]	0.67 [0.35, 1.28]
Kicked 1–5 times	0.79 [0.39, 1.63]	0.79 [0.39, 1.62]	0.79 [0.39, 1.60]	0.78 [0.39, 1.58]
CSA-Forced 6+ times (ref.)				
Never Forced	0.29 [0.04, 1.96]	0.78 [0.27, 2.28]	0.75 [0.26, 2.18]	0.74 [0.26, 2.15]
Forced 1–5 times	0.29 [0.03, 2.81]	1.17 [0.37, 3.70]	1.13 [0.35, 3.57]	1.12 [0.35, 3.51]
CSA-Touched 6+ times (ref.)				
Never Touched	1.02 [0.39, 2.71]	0.58 [0.14, 2.42]	1.01 [0.39, 2.61]	1.01 [0.39, 2.58]
Touched 1–5 times	1.26 [0.46, 3.45]	0.62 [0.11, 3.41]	1.23 [0.46, 3.31]	1.23 [0.46, 3.29]
CEA-Felt Hurt 6+ times (ref.)				
Never Felt Hurt	0.93 [0.65, 1.34]	0.94 [0.65, 1.34]	0.88 [0.41, 1.92]	0.94 [0.66, 1.35]
Felt Hurt 1–5 times	1.03 [0.73, 1.44]	1.03 [0.73, 1.44]	0.70 [0.29, 1.68]	1.03 [0.73, 1.44]
CEA- Felt Unloved 6+ times (ref.)				
Never Felt Unloved	0.70 [0.46, 1.07]^†^	0.70 [0.46, 1.07]^†^	0.70 [0.46, 1.07]^†^	0.75 [0.33, 1.68]
Felt Unloved 1–5 times	0.94 [0.63, 1.41]	0.95 [0.63, 1.42]	0.95 [0.64, 1.43]	0.57 [0.18, 1.77]
Community Belonging	0.60 [0.34, 1.05]^†^	0.71 [0.45, 1.12]	0.82 [0.68, 0.99]^*^	0.86 [0.65, 1.12]
Belonging*Forced 6+ times (ref.)				
Belonging*Never Forced	1.45 [0.82, 2.57]			
Belonging*Forced 1–5 times	1.69 [0.83, 3.44]			
Belonging*Touched 6+ times (ref.)				
Belonging*Never Touched		1.22 [0.77, 1.95]		
Belonging*Touched 1–5 times		1.29 [0.72, 2.31]		
Belonging*Hurt 6+ times (ref.)				
Belonging*Never Hurt			1.03 [0.80, 1.32]	
Belonging*Hurt 1–5 times			1.16 [0.85, 1.58]	
Belonging*Unloved 6+ times (ref.)				
Belonging*Never Unloved				0.98 [0.73, 1.32]
Belonging*Unloved 1–5 times				1.22 [0.80, 1.84]
*∆* *F*	1.09	0.41	0.47	0.86

*Note.* Ref. = reference group; CPA = childhood physical abuse; CSA = childhood sexual abuse; CEA = childhood emotional abuse. All models controlled for age, age^2^, income, sex, sexual orientation, marital status, education, region, ethnoracial identity, place of birth, and population centre. Covariates were omitted from the table to improve clarity.

^†^ *p* ≤.10, * *p* ≤.05, ** *p* ≤.01, *** *p* ≤.001.

**Table 5:** Marginal Mean Comparisons of the Likelihood of Cyberbullying Victimization Between the ‘Never-Abuse’ and ‘High- Abuse’ Groups for Each Childhood Abuse Variable, Across Levels of Community Belonging.

	Level of Community Belonging			
Comparison Groups	Very Weak	Somewhat Weak	Somewhat Strong	Very Strong
Never Slapped vs. Slapped 6+ times	10.0%	8.6%	6.6%	4.7%
Never Pushed vs. Pushed 6+ times	17.1%	10.6%	8.3%	6.4%
Never Kicked vs. Kicked 6+ times	10.9%	10.8%	14.6%	13.2%
Never Forced vs. Forced 6+ times	24.3%	14.0%	6.3%	1.7%
Never Touched vs. Touched 6+ times	15.6%	10.4%	6.5%	3.2%
Never Emotionally Hurt vs. Hurt 6+ times	6.9%	6.6%	5.1%	4.2%
Never Felt Unloved vs. Unloved 6+ times	8.8%	8.3%	7.4%	6.5%

## Discussion

Past research shows that experiencing childhood abuse predicts revictimization later in life [24]. However, studies examining CBV have focused on adolescents and young adults, rather than adults. The current study addressed this gap by examining whether community belonging is associated with a lower risk of experiencing CBV among adults with histories of physical, sexual, or emotional childhood abuse. H1 was partially supported: the ‘Never-abuse’ categories showed trivial-to-small effects for lower CBV risk in adulthood compared to the ‘High-abuse’ categories, although these differences were not statistically significant. H2 was supported, as community belonging negatively predicted adult CBV. However, no significant moderation effects of community belonging were found, so H3 was not supported. Despite the nonsignificant moderation effects, a trend was observed: the marginal means differences in adult CBV between the ‘Never-abuse’ groups and ‘High-abuse’ groups were large at weaker belonging and small at stronger belonging (H3). These trends will be discussed as exploratory.

### Nonsignificant or underpowered?

Statistical nonsignificance can be thought to appear in two different flavours: a real relationship is there but there was inadequate power to detect it (i.e., Type II error), or there is no relationship between the variables, and the nonsignificance simply reflects that. While the statistical results were inconsistent with our moderation hypotheses, as can be seen by Figs 1–7, the pattern of results is often consistent with what we predicted. Specifically, the ‘gap’ between the ‘High-abuse’ groups and the ‘Never-abuse’ groups was the largest at the lowest end of community belonging and was the smallest at the highest end of community belonging. This pattern is present in Fig 1 (10.0% shrank to 4.7%), Fig 2 (17.1% shrank to 6.4%), Fig 4 (24.3% shrank to 1.7%), and Fig 5 (15.6% shrank to 3.2%). In contrast, if the moderation hypothesis was simply wrong, then we would expect to see no shrinkage in some values, growth in others, and no changes in others. Despite a large sample size (*n* ≈ 14,000), our models were underpowered due to the infrequency of respondents reporting high levels of abuse *and* weak community belonging. The rarity of these respondents contributed to the large error estimation, which drove nonsignificance.

### Interpreting complex patterns

The most pronounced differences in adult CBV risk across increasing levels of community belonging were observed for sexual abuse. The differences in adult CBV risk between these ‘High-abuse’ and 'Never-abuse' groups were initially the largest at weak community belonging. These gaps closed to near parity at strong belonging, suggesting that CSA has a greater impact on adult CBV risk when belonging is weak. CSA experiences can be deeply damaging and often result in psychological and social impacts, including lower self-esteem [55], feelings of shame [56], mistrust, and poorer social connections [57]. Community belonging may foster trust, emotional support, and social involvement, reducing the risk of adult CBV.

A similar pattern emerged for CPA behaviours, including slapping and pushing, where the likelihood of CBV was higher for both the ‘Never-abuse’ groups and ‘High-abuse’ groups at weaker levels of belonging and lower at stronger belonging. However, this pattern did not hold for the most extreme form of physical abuse, being kicked, bit, punched, choked, or attacked. These results suggest that community belonging can help mitigate the risk of adult CBV following less severe CPA but is less effective after more extreme abuse. This aligns with research showing that the protective effect of social support was weaker for individuals who experienced multiple types of childhood maltreatment [58] or more severe abuse [59]. Because severe childhood abuse is associated with pervasive psychological harm [60], the protective effects of belonging may not be sufficient to protect against its impact. These insights have implications for interventions, as severe forms of physical abuse may require additional evidence-based approaches beyond community belonging.

### Disaggregating abuse typologies

Our study explicitly disaggregated forms of childhood abuse to reduce issues with heterogeneity. These efforts were largely justified given the contrast in CSA, CPA, and CEA results. Specially, relative to CSA and CPA experiences, the CEA behaviours did not show the predicted pattern of moderation where increasing community belonging had a more profound buffering effect for abused groups (Figs 6 and 7). While social connections through community belonging may reduce the negative self-perceptions [6], it is likely that the emotional abuse variables capture a greater degree of heterogeneity than either physical abuse or sexual abuse. Emotional abuse can be more subjective and context-dependent than physical and sexual abuse, which may contribute to variability in how respondents interpret and report such experiences. Specifically, if a person ever felt unloved or emotionally hurt by any caregiver prior to the age of 15, then they would respond in the affirmative to this question. As can be seen in Table 1, emotional abuse is the most common form, which makes sense as emotional harm could theoretically be achieved unintentionally, whereas both physical and sexual abuse are commonly deliberate acts. It may be that the individuals reporting CEA have a greater diversity in childhood upbringing than individuals reporting CPA and CSA, which may adulterate the comparison groups. In any event, we would suspect that the nonsignificance of the interaction terms for CEA reflect a genuinely absent finding (or one that has to be more carefully teased apart), rather than a Type II error.

### Fitting-in with the extant literature

The direction of these findings is consistent with previous literature showing that higher levels of childhood abuse predicted greater vulnerability to CBV compared to those without a history of abuse [2,4,6]. These findings also align with research indicating that childhood adversity is associated with high-risk online behaviours and smart phone addictions across younger age groups [35], which may increase the risk of CBV. Moreover, childhood abuse can negatively impact self-esteem, trust, and emotion regulation, which can impair healthy social relationships and increase susceptibility to further victimization [3,6,32–34]. From an ecological perspective, these individual vulnerabilities may interact with broader factors, such as unsupportive peer networks, limited community resources, or societal tolerance of online aggression, to increase CBV risk in adulthood. Additionally, children exposed to violence may become more accepting of being bullied, as suggested by social learning theory [61], thereby increasing their vulnerability to CBV [2]. However, in the current study, the associations between the ‘Low-to-moderate abuse’ groups and CBV were less consistent, with some abusive behaviours showing slightly higher, but nonsignificant, odds of experiencing adult CBV compared to the ‘High-abuse’ categories. Comparing the current finding to previous studies is challenging, as past research on childhood abuse and CBV often examined cumulative abuse scores [1,3,7], whereas we examined individual groups of abusive behaviours. Cumulative abuse scores often provide a more comprehensive measure, as abusive behaviours tend to co-occur [19], which may amplify vulnerability to CBV. The current pattern of findings, although nonsignificant, align with past research on the protective benefits of community belonging following childhood abuse [16,46]. The results also relate to the concept of compounding risk factors, as both childhood abuse [1,6] and weak community belonging [27,62] are independent risk factors for CBV, and combined, they magnify the effect. However, individuals who reported experiences of being abused but who had strong community belonging showed a more gradual slope in adult CBV risk than those with the same level of abuse and low community belonging.

### Limitations, future directions, and conclusion

Some important limitations should be considered. First the GSS is a self-report survey, which introduces recall bias to the childhood abuse questions, as participants are asked to report experiences that may have occurred decades earlier. Memory for emotionally salient or traumatic events may be influenced by time and individual’s mental health, which can result in either underreporting or overreporting of such events [63]. Second, the study examined a sample of the Canadian population, which may limit the generalizability of the results to other populations. Third, the cross-sectional data prevents causation from being inferred. Although the temporal sequence among variables is clear, all variables were measured at one time point; therefore, factors such as memory bias, current mood, or the timing of other life changes may have influenced participant reporting and observed effects. Fourth, the high abuse categories had small sample sizes resulting in an underpowered analysis and an increased risk of Type II error. Detecting interaction effects requires much larger samples than detecting main effects to obtain adequate statistical power [64]. In the current study, the interactions between higher frequencies of abuse and community belonging were estimated with a high error, potentially obscuring key nuances. Despite these limitations, we had a large, nationally representative sample and conducted a granular analysis of childhood abuse, which examined three categories of abuse and distinct behaviours or feelings within each type.

The current study contributes to understanding the association between childhood abuse, community belonging, and CBV in adulthood in a Canadian context. The findings highlight the importance of fostering strong community connections, which could include programs, support groups, volunteer engagement, and workplace or school initiatives, as a potential method to reduce the risk of cyberbullying among adults who have experienced childhood abuse. These findings have implications for clinicians, social workers, public health organizations, schools, and society at large, who can support initiatives that foster safe community environments. Future research should examine the association between childhood abuse, community belonging, and CBV by age categories to assess whether the protective effects of belonging vary across developmental stages. Additionally, more work should be done to explore whether experiencing low-to-moderate levels of abuse consistently predicts higher odds of CBV than experiencing high levels of abuse.

The mechanism connecting childhood abuse and adult CBV is likely complex. Childhood abuse is associated with emotion dysregulation, mistrust of others, impaired social skills, and negative perceptions of the self [3,6,32]. Community belonging may offer broad social networks that provide psychological support and reduce loneliness, enhance awareness on cyberbullying, and bolster self-worth by fostering a sense of identity, self-esteem, and social-emotional skills [4,65]. Additionally, individuals with strong community ties may participate in more community events, leaving them with limited time for online activities. These social and personal factors may work together to reduce the risk of adult CBV after childhood abuse.
